# Cholinesterase inhibitors do not alter the length of stay in nursing homes among patients with Alzheimer’s disease: a prospective, observational study of factors affecting survival time from admission to death

**DOI:** 10.1186/s12883-016-0675-3

**Published:** 2016-08-31

**Authors:** Carina Wattmo, Elisabet Londos, Lennart Minthon

**Affiliations:** 1 Clinical Memory Research Unit, Department of Clinical Sciences, Malmö, Lund University, SE-205 02 Malmö, Sweden; 2 Memory Clinic, Skåne University Hospital, SE-205 02 Malmö, Sweden

**Keywords:** Alzheimer’s disease, Cholinesterase inhibitors, Activities of daily living, Cognition, Community-based services, Survival time in nursing homes, Sex, Predictors, Longitudinal study

## Abstract

**Background:**

The survival time in nursing homes (NHs) in Alzheimer’s disease (AD) might be affected by sociodemographic/clinical characteristics, rate of disease progression, and use of specific medications and community-based services. Whether different aspects of cholinesterase inhibitor (ChEI) therapy modify time spent in NHs is unclear. Therefore, we examined the relationship between these potential predictors and survival time in NHs.

**Methods:**

This prospective, multicenter study of ChEI treatment in clinical practice included 220 deceased patients clinically diagnosed with mild-to-moderate AD who were admitted to NHs during the study. Cognitive and activities of daily living (ADL) performance, ChEI dose, and amount of services used/week were evaluated every 6 months over 3 years. Dates of nursing-home placement (NHP) and death were recorded. Variables that determined survival time in NHs were analyzed using general linear models.

**Results:**

The mean survival time in NHs was 4.06 years (men, 2.78 years; women, 4.53 years; *P* < 0.001). The multivariate model showed that a shorter stay in NHs was associated with the interaction term male living with a family member, use of antihypertensive/cardiac therapy or anxiolytics/sedatives/hypnotics, and worse basic ADL at NHP, but not with age or cognitive and instrumental ADL capacities.

**Conclusions:**

Increased community-based care did not reduce the survival time in NHs among individuals with AD. Men living with family spent significantly less time in NHs compared with the corresponding women, which suggests that the situation of female spouses of AD patients may need attention and possibly support. There was no indication that different aspects of ChEI therapy, e.g., drug type, dose, or duration, alter survival time in NHs.

## Background

Alzheimer’s disease (AD) is a progressive, neurodegenerative disorder that initially exhibits cognitive symptoms, such as impaired memory, disorientation, and reduced executive ability, which are accompanied by deterioration in activities of daily living (ADL) [1]. AD is the most common form of dementia and its prevalence increases exponentially with age. About 10 % of people over 65 years will experience some type of dementia, whereas one-third of those over 85 will be affected by this condition [2].

The need of assistance starts early in patients with AD, and deficits in instrumental ADL tasks have been reported even in the stages of mild cognitive impairment and mild AD [3, 4]. Their care needs develop constantly and about half of the individuals with dementia need help with personal care, whereas the remaining half will need such help over time [5]. This results in an increasing demand for community-based services (e.g., home help and adult day care) and nursing-home placement (NHP), the costs of which rise dramatically with advancing disease severity [6]. Currently, about 30 %–50 % of people with dementia in high-income countries receive care in nursing homes (NHs) [5]. The annual cost of NH care in Sweden for one recipient in 2014 was SEK 610,000 (~USD 69,600, ~EUR 63,700) [7]. In middle-income countries in particular, an increased demand for high-quality care is expected. It is necessary for health authorities to plan and allocate resources for different types of dementia care and special accommodation [5].

The symptomatic treatment mainly recommended for mild-to-moderate AD is cholinesterase inhibitor (ChEI; donepezil, rivastigmine, and galantamine) therapy. ChEIs inhibit the degradation of the neurotransmitter acetylcholine by acetylcholinesterase, resulting in increased levels of acetylcholine in the synaptic cleft and increased availability for receptor absorption. This sustains cholinergic transmission and improves communication between neurons [8]. The level of short-term response to ChEI and the long-term outcomes vary among persons with AD [9]. No difference in effect has been detected among the three ChEI agents [10, 11]. However, higher ChEI doses, regardless of drug agent, were associated with better longitudinal cognitive and functional capacities [11–13]. Significant associations between a higher dose of ChEI and lower amount of home-help services [14], postponed need for NHP [15, 16], and longer life-span [17] have also been observed. Moreover, we reported recently that a longer ChEI treatment duration was independently related to longer survival in AD [17]. Whether different aspects of ChEI therapy alter the survival time in NHs has not been investigated.

Few previous studies have described the length of time spent in NHs for AD patients exclusively. Two American studies observed a median of 33 and 38.57 months, respectively [18, 19], although the factors that might affect this parameter were not addressed. However, predictors of mortality in NH residents with AD have been reported, such as male sex, older age, and functional disability. The severity of dementia and the impact of comorbid illnesses yielded inconsistent results [20].

Here, we aimed to identify sociodemographic and clinical factors, including various aspects of ChEI treatment, AD progression, and use of community-based services, that might predict survival time in NHs.

## Methods

### Participants and setting

The Swedish Alzheimer Treatment Study (SATS) began in 1997 to investigate different aspects of the effectiveness of long-term ChEI therapy in a routine clinical setting. SATS is a 3-year, open-label, observational, nonrandomized, multicenter study that was described previously [4, 11, 13, 14, 16, 17]. Before inclusion, all patients underwent a thorough clinical investigation including medical history, physical and neurological examinations, cognitive evaluations, laboratory tests, and cerebral computed tomography to rule out other causes of dementia. Additionally, in some centers, the individuals were investigated further through cerebrospinal fluid tap, measurement of regional cerebral blood flow (Cortexplorer using 133-Xenon inhalation or single-photon emission computed tomography), electroencephalography, and neuropsychological tests. Outpatients aged ≥40 years who had been clinically diagnosed with dementia, as defined by the Diagnostic and Statistical Manual of Mental Disorders, 4th edition (DSM-IV) [21], and with possible or probable AD according to the criteria of the National Institute of Neurological and Communicative Disorders and Stroke and the Alzheimer’s Disease and Related Disorders Association (NINCDS-ADRDA) [22] were considered for inclusion in the SATS. The participants were diagnosed by specialists in dementia disorders. Moreover, the individuals had to be living at their own home at the time of AD diagnosis, have a responsible caregiver, and be assessable using the Mini-Mental State Examination (MMSE) [23] at the start of ChEI treatment (baseline). The 1,258 participants enrolled here were prospectively recruited from 14 memory clinics across Sweden; most of them were in the mild-to-moderate stages of AD. All 220 deceased individuals who were admitted to NHs during the SATS and had MMSE baseline scores from 10 to 26 were included in this study.

The patients were evaluated in a structured follow-up program, which assessed cognition, instrumental and basic ADL performance, and community-based service utilization (home-help services and adult day care) before the start of ChEI therapy and semi-annually over 3 years. After inclusion and baseline evaluations, the participants were prescribed ChEIs according to the approved product recommendations. The choice of drug and dosage for each individual was left entirely up to the dementia specialist’s discretion and professional judgment, i.e., the standard routine in clinical practice. The ChEI dose was recorded after 2 months of treatment, and every 6 months after the baseline. Trained dementia nurses obtained the ADL assessment and the amount of service used/week (if any) from an interview with the caregiver (usually the spouse or an adult child). Medications other than ChEIs were permitted, with the exception of memantine, and were documented at baseline. If memantine was initiated, the patient dropped out from the SATS at that time point.

The dementia specialist obtained the estimated age at the onset of AD from an interview with the caregiver. The date of NHP was obtained from medical records, and NHP was defined as the permanent entry to a licensed skilled-nursing facility with 24 h care; i.e., the residents were not discharged home, and rehabilitative or respite care was excluded. If hospitalization occurred before NHP, the date of hospital admission was used. The date of death (up until December 31, 2014) was obtained from the Swedish population register (Swedish Tax Agency).

### Assessment scales

Cognitive ability was evaluated using the MMSE scale (0–30 points), in which a lower score indicates more impaired cognition, and the Alzheimer’s Disease Assessment Scale—cognitive subscale (ADAS-cog) (0–70 points) [24], in which a lower score indicates better cognitive status. Functional capacity was assessed using the Instrumental Activities of Daily Living (IADL) scale [25], comprising eight items: telephone use, shopping, food preparation, housekeeping, doing laundry, mode of transportation, responsibility for own medications, and ability to handle finances. Each item was scored from 1 (no impairment) to 3–5 (severe impairment), allowing a total range of 8–31 points. Basic ADL were measured using the Physical Self-Maintenance Scale (PSMS) [25], comprising six items: toilet use, feeding, dressing, grooming, physical ambulation, and bathing. Each item was scored from 1 (no impairment) to 5 (severe impairment), giving a total range of 6–30 points.

### Statistical analyses

The IBM Statistical Package for the Social Sciences (SPSS) for Windows (version 22.0; IBM Corporation, Armonk, NY, USA) was used to perform statistical analyses. The level of significance was defined as *P* < 0.05 if not otherwise specified, and all tests were two-tailed. Parametric tests were used because of the large sample size and the approximately normally distributed continuous variables. A one-way analysis of variance (ANOVA) was performed to compare the differences between the means obtained for three or more independent groups, such as the interaction effect of sex by living status, and groups divided according to PSMS score. A *t* test was used to analyze two independent groups, e.g., sex, living status, and use of specific medications. Pearson’s correlation coefficient was calculated to investigate any linear associations between continuous predictors, such as survival time in NHs and age, cognitive or functional performance, or number of concomitant medications.

#### General linear models

The multivariate approach of general linear models was used in this study because of the large sample of deceased participants for whom dates of NHP were available; thus, no patients were censored. General linear models were used (1) to simultaneously estimate the possible effect of the sociodemographic and clinical predictors mentioned below on the dependent variable “survival time in NHs” (in years) and (2) to explore the effect of the use of community-based services on time spent in NHs by adding those factors to the first model. Nonsignificant variables (*P* > 0.05) were eliminated using the backward stepwise approach. The hierarchical principle was observed in these analyses; terms that appeared in interactions were not considered for elimination.

Well-known risk factors, e.g., sex, age at NHP, the clinician’s estimation of duration of AD, years of education, number of apolipoprotein E (APOE) ε4 alleles, and solitary living at NHP (no/yes), were included in the first general linear model. Measures of AD severity and progression, i.e., cognitive (MMSE score only because of its strong linear correlation with the ADAS-cog), instrumental, and basic ADL abilities at NHP and their rates of change/year before NHP, were also included as independent variables. Comorbidity was investigated using the number of concomitant medications at baseline as a potential predictor, as well as the presence of specific medications (no/yes for each group): antihypertensive/cardiac therapy, antidiabetic drugs, asthma medication, thyroid therapy, lipid-lowering agents, estrogens, nonsteroidal anti-inflammatory drugs (NSAIDs)/acetylsalicylic acid, antidepressants, antipsychotics, and anxiolytics/sedatives/hypnotics. The impact of ChEI therapy during the SATS was analyzed by including the different drug agents (coded as dummy variables), ChEI dose, and treatment duration (in months) in the model. A second general linear model, together with the abovementioned variables, included the use of home-help services (h/week) and adult day care (days/week) at NHP and annual changes in the volume of these services before NHP.

The change in score (MMSE, IADL, and PSMS) or in the amount of services/week from baseline to the individual’s last evaluation before NHP was divided by the number of months between these evaluations and multiplied by 12. To facilitate comparisons between the MMSE, IADL, and PSMS scales, changes in the scores reflected as positive values should be interpreted as indicating improvement and those reflected as negative values as indicating worsening. Most participants did not receive community-based services at baseline; therefore, these possible predictors were treated as categorical variables because of their skewed distributions.

The ChEI dose could vary during the treatment period for an individual patient and between patients. Therefore, the mean dose used during the entire follow-up period was calculated for each individual. Furthermore, to obtain a similar metric of maximum dosage percentage for the three ChEI agents, the mean dose was divided by the maximum recommended dose for each drug agent; i.e., 10 mg for donepezil, 12 mg for rivastigmine (oral therapy), and 24 mg for galantamine.

## Results

### Survival time in nursing homes

**Fig. 1:**
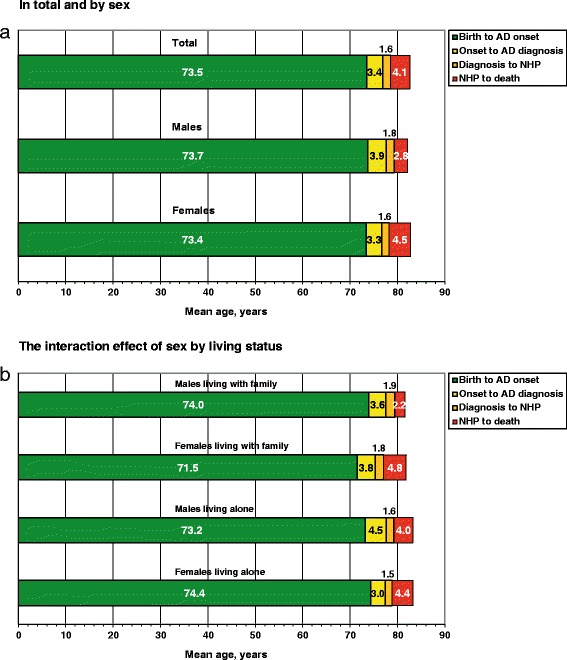
Time course of events in the SATS according to sociodemographic characteristics. Mean age at AD onset, illness duration, time from diagnosis (start of cholinesterase inhibitor treatment) to NHP, and survival time in NHs. **a.** In total and according to sex. Females with AD spent on average 1.75 more years (21 months) in NHs than did males (*P* < 0.001). No sex-based difference was observed regarding age at AD onset, illness duration, age at diagnosis, time between diagnosis and NHP, age at NHP, and age at death. **b.** The interaction effect of sex with living status. Females living with family showed a trend toward a younger age at the onset of AD symptoms than did the other groups (*P* = 0.074). Solitary living males with AD exhibited a trend toward a significantly longer illness duration compared with females living alone (*P* = 0.055). Males living with a family member had longer time from AD diagnosis to NHP than did solitary living females (*P* = 0.022). Moreover, males living with family spent a mean of ~2–2.5 years less time in NHs compared with the other groups (*P* < 0.001). No significant differences were detected regarding age at diagnosis, age at NHP, and age at death. AD, Alzheimer’s disease; NH, nursing home; NHP, nursing home placement; SATS, Swedish Alzheimer Treatment Study

The sociodemographic and clinical characteristics of the 220 SATS participants at the start of ChEI treatment (baseline) and at NHP are shown in Table 1. Overall, their survival time in NHs was (mean (95 % confidence interval (CI)) 4.06 (3.69–4.43) years. Females with AD spent a longer time in NHs than did males, 4.53 (4.09–4.96) years vs 2.78 (2.19–3.38) years (t_218_ = −4.71, *P* < 0.001). Patients living alone at NHP demonstrated a trend toward a significantly longer survival time in NHs vs those living with family, 4.35 (3.87–4.83) years vs 3.67 (3.10–4.25) years (t_218_ = −1.79, *P* = 0.075). The potential interaction effect of sex by living status was also analyzed. Post hoc tests (Bonferroni) showed that males living with a family member at NHP, *n* = 39, spent a shorter time in NHs, on average (2.15 (1.48–2.83) years) than did patients in the other groups: females living with family, *n* = 55, 4.75 (4.00–5.50) years; males living alone, *n* = 20, 4.00 (2.96–5.05) years; and females living alone, *n* = 106, 4.41 (3.87–4.95) years, F_3,216_ = 8.66, *P* < 0.001. Women living with a family member had a lower mean MMSE score at NHP vs females who lived alone, 15.7 (13.8–17.6) points vs 18.4 (17.5–19.3) points; however, their cognitive status did not differ from that of the other two groups: males living with family, 16.8 (14.9–18.7) points and males living alone, 19.4 (16.9–21.8) points, F_3,216_ = 3.70, *P =* 0.013. The solitary-living individuals exhibited a better mean IADL score at NHP, regardless of sex: males, 21.5 (19.3–23.7) points and females, 21.5 (20.5–22.4) points vs those living with family: males, 24.6 (23.3–25.8) points and females, 24.2 (23.0–25.4) points, F_3,216_ = 7.26, *P <* 0.001. No significant differences regarding age or basic ADL at NHP, or number of concomitant medications at baseline were found among the four groups. Figure 1 a–b illustrates the patients’ age at onset of AD, age at baseline (shortly after AD diagnosis), age at NHP, and age at death, on average, overall, and according to sex, as well as the interaction effect of sex by living status.

**Table 1:** Sociodemographic and Clinical Characteristics (*n* = 220)

	At the start of ChEI treatment	At nursing-home placement^a^
Variable	*n*/%	*n*/%
Female sex	161/73 %	na
APOE genotype, (*n* = 208)		na
No ε4 alleles	57/27 %	
One ε4 allele	118/57 %	
Two ε4 alleles	33/16 %	
Solitary living	116/53 %	126/57 %
Antihypertensive/cardiac therapy	79/36 %	
Antidiabetics	9/4 %	
Asthma medication	9/4 %	
Thyroid therapy	19/9 %	
Lipid-lowering agents	15/7 %	
Estrogens	17/8 %	
NSAIDs/acetylsalicylic acid	63/29 %	
Antidepressants	71/32 %	
Antipsychotics	14/6 %	
Anxiolytics/sedatives/hypnotics	41/19 %	
Variable	Mean ± standard deviation	
Estimated age at onset of AD, years	73.5 ± 6.9	na
Estimated duration of AD, years	3.4 ± 2.6	5.1 ± 2.7
Age, years	76.9 ± 5.9	78.6 ± 5.8
Education, years	9.2 ± 2.3	na
MMSE score	20.1 ± 4.0	17.5 ± 5.7
ADAS-cog score (0–70)	24.0 ± 9.7	29.2 ± 12.5
IADL score	18.3 ± 5.1	22.7 ± 4.7
PSMS score	8.2 ± 2.8	10.5 ± 3.9
Number of concomitant medications^a^	3.0 ± 2.4	
Home-help service, h/week	6.6 ± 5.4 (*n* = 56)	5.7 ± 6.5 (*n* = 180)
Adult day care, days/week	3.2 ± 1.7 (*n* = 23)	3.0 ± 1.4 (*n* = 82)
Mean dose of ChEI during the SATS, mg		
Donepezil (*n* = 138)	6.8 ± 1.8	na
Rivastigmine (*n* = 37)	5.7 ± 1.9	na
Galantamine (*n* = 45)	15.1 ± 3.2	na
Time to nursing home placement from the start of ChEI therapy, months	19.5 ± 10.0	na
Age at death, years	82.6 ± 6.0	na

*AD* Alzheimer’s disease, *ADAS-cog* Alzheimer’s Disease Assessment Scale – cognitive subscale, *APOE* apolipoprotein E, *ChEI* cholinesterase inhibitor, *IADL* Instrumental Activities of Daily Living scale, *MMSE* Mini-Mental State Examination, *na* not applicable, *NSAIDs* nonsteroidal anti-inflammatory drugs, *PSMS* Physical Self-Maintenance Scale, *SATS* Swedish Alzheimer Treatment Study

^a^Concomitant medications were not recorded at the postbaseline visits

**Fig. 2:**
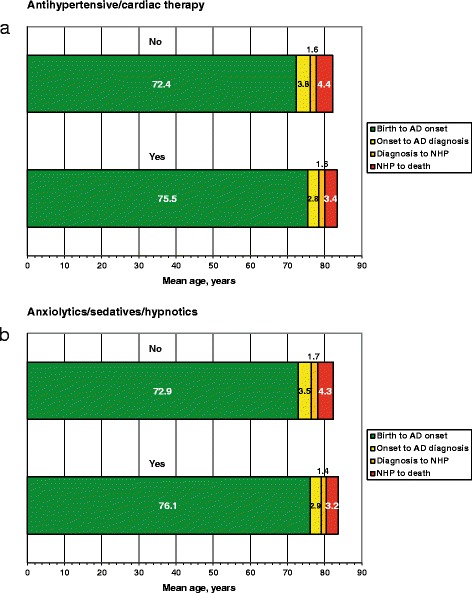
Time course of events in the SATS according to concomitant medications. Mean age at AD onset, illness duration, time from diagnosis (start of cholinesterase inhibitor treatment) to NHP, and survival time in NHs. **a** Antihypertensive/cardiac therapy. The participants without antihypertensive/cardiac therapy were younger at the onset of AD symptoms (*P* < 0.001), had a longer illness duration (*P* = 0.004), and were younger at the time of diagnosis (*P* = 0.003) and at NHP (*P* = 0.002) compared with the individuals who received these medications. The survival time in NHs was, on average, 1 year longer for patients with no antihypertensive/cardiac therapy (*P* = 0.007). No significant difference was found regarding time between diagnosis and NHP, and age at death. **b** Anxiolytics/sedatives/hypnotics. The participants without these medications were younger at the onset of AD (*P =* 0.008), at the time of diagnosis (*P* = 0.011), and younger at NHP (*P* = 0.018) compared with the individuals who received anxiolytics/sedatives/hypnotics. The survival time in NHs was a mean of 1 year shorter for users of these medications (*P* = 0.032). No significant difference was observed regarding illness duration, time between diagnosis and NHP, and age at death. AD, Alzheimer’s disease; NH, nursing home; NHP, nursing home placement; SATS, Swedish Alzheimer Treatment Study

The mean survival time in NHs was shorter for individuals treated with antihypertensive/cardiac therapy at baseline, 3.39 (2.78–3.99) years vs 4.44 (3.98–4.89) years, t_218_ = 2.74, *P* = 0.007, and for those using anxiolytics/sedatives/hypnotics, 3.22 (2.45–4.00) years vs 4.25 (3.84–4.67) years, t_218_ = 2.16, *P* = 0.032. There was a trend toward significance (*P* = 0.060) for the participants (*n* = 9) treated with antidiabetics at the start of ChEI treatment to exhibit also a shorter time spent in NHs. Users of antihypertensive/cardiac therapy were older, on average: 80.0 (79.0–81.0) years vs 77.7 (76.7–78.8) years, t_218_ = −3.09, *P* = 0.002; and had a somewhat higher MMSE score at NHP, 18.6 (17.5–19.7) points vs 16.9 (15.9–17.9) points, t_218_ = −2.17, *P* = 0.031, but similar functional capacity compared with the nonusers. Users of anxiolytics/sedatives/hypnotics were also older at NHP, 80.5 (78.8–82.1) vs 78.1 (77.3–79.0) years, t_218_ = −2.39, *P* = 0.018, but their cognitive and functional performance did not differ from those of the nonusers. Figure 2 a–b illustrates the patients’ age at onset of AD, at baseline, at NHP, and at death, on average, according to use of antihypertensive/cardiac therapy and of anxiolytics/sedatives/hypnotics.

A shorter survival time in NHs exhibited weak linear associations with older age at NHP (*r* = −0.136, *P* = 0.043), lower basic ADL at NHP (PSMS score; *r* = −0.183, *P* = 0.007), and a higher number of medications at baseline (*r* = −0.170, *P* = 0.011).

### Factors that affect survival time in nursing homes

**Fig. 3:**
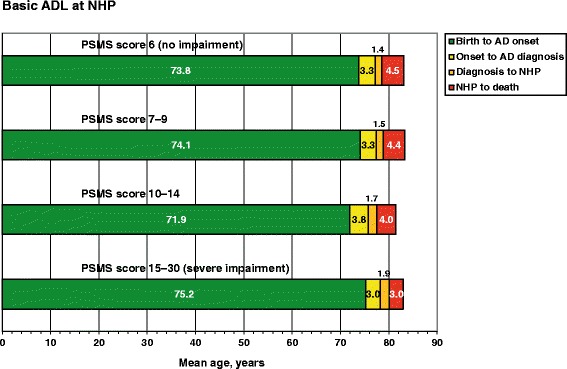
Time course of events in the SATS according to the basic ADL capacity at NHP. Mean age at AD onset, illness duration, time from diagnosis (start of cholinesterase inhibitor treatment) to NHP, and survival time in NHs, in the four groups of participants (PSMS score: 6, 7–9, 10–14, and 15–30). The individuals who exhibited a PSMS score of 10–14 at NHP spent about 0.5 year shorter time in NHs and those who had a PSMS score of ≥15 at NHP spent an average of ~1.5 years shorter time in NHs compared with patients who had a PSMS score of 6 (no impairment) at NHP (*P* = 0.045). More impaired basic ADL at NHP and longer time between diagnosis and NHP showed a significant relationship (*P* = 0.048). No differences were found regarding age at AD onset, illness duration, age at diagnosis, age at NHP, and age at death among the four groups. AD, Alzheimer’s disease; ADL, activities of daily living; NH, nursing home; NHP, nursing home placement; PSMS, Physical Self-Maintenance Scale; SATS, Swedish Alzheimer Treatment Study

General linear models using the time between NHP and death as the dependent variable were built to identify the sociodemographic and clinical factors that influenced the individuals’ time spent in NHs. The degree of explanation of the variance in the model was moderate (R = 0.458, R^2^ = 0.210, *P* < 0.001). The multivariate model and significant predictors are presented in Table 2. A shorter survival time in NHs was related to use of antihypertensive/cardiac therapy, use of anxiolytics/sedatives/hypnotics, worse basic ADL at NHP, and the interaction term male living with family. Figure 3 shows the mean age at onset of AD, at baseline, at NHP, and at death in the four groups of patients, depending on basic ADL at NHP (PSMS score, 6: no impairment; 7–9, 10–14, and 15–30: severe impairment).

**Table 2:** Factors that Affected the AD patients’ Survival Time in Nursing Homes, years (Final General Linear Model)

Significant predictors^a^	β	95 % CI	*P* value
Intercept	2.069	0.521, 3.618	0.009
Sex by living status^b^			
Females living with family	2.489	1.427, 3.551	<0.001
Females living alone	2.185	1.239, 3.130	<0.001
Males living alone	1.590	0.208, 2.972	0.024
Antihypertensive/cardiac therapy (no = 0, yes = 1)	−0.884	−1.599, −0.170	0.016
Anxiolytics/sedatives/hypnotics (no = 0, yes = 1)	−1.030	−1.908, −0.153	0.022
PSMS score at NHP	−0.119	−0.208, −0.030	0.009

β values were unstandardized and are expressed per 1 unit increase for continuous variables, and for the condition present for dichotomous variables

Apolipoprotein E genotype, duration of AD, age at NHP, years of education, cognitive or instrumental ADL abilities at NHP, cholinesterase inhibitor agent, dose or duration of treatment, other concomitant medications (antidiabetics, asthma medication, thyroid therapy, lipid-lowering agents, estrogens, nonsteroidal anti-inflammatory drugs/acetylsalicylic acid, antidepressants, or antipsychotics), the amount of home-help services, or adult day care at NHP or the annual mean changes in these services before NHP were not significant. There were no associations observed between survival time in nursing homes and the level of cognitive or functional response to cholinesterase inhibitors after 6 months of treatment, or rate of disease progression before NHP

*AD* Alzheimer’s disease, *CI* confidence interval, *NHP* nursing home placement, *PSMS* Physical Self-Maintenance Scale

^a^Degree of explained variance, R = 0.458, R^2^ = 0.210, *P* < 0.001

^b^Males living with family were the reference category

## Discussion

In this longitudinal AD study performed in a routine clinical practice setting, we found that the mean survival time in NHs was about 4 years. Females spent 1.75 years (21 months) more in NHs than males. The general linear model showed that a shorter stay in NHs was independently associated with the interaction term male living with a family member, use of antihypertensive/cardiac therapy, use of anxiolytics/sedatives/hypnotics, and lower basic ADL capacity (but not IADL) at NHP. Cognitive ability did not affect the survival time in NHs. Males living with family spent ~2–2.5 years less in NHs compared with the other groups, despite the absence of significant differences regarding age, disease severity, or number of concomitant medications. Users of antihypertensive/cardiac therapy or anxiolytics/sedatives/hypnotics spent 1 year less, on average, in NHs than nonusers. Impairment in basic ADL might imply a shorter time spent in NHs of up to 1.5 years, depending on severity. No relationships between different aspects of ChEI treatment and survival time in NHs were detected.

The mean time spent in NHs (4.06 years) found in the present AD study is consistent with the 4.3 years observed for demented persons reported by the National Board of Health and Welfare, Sweden [26]. A recent German study of dementia [27] described a similar survival time in NHs for females (mean, 52.5 months), and a slightly longer time for males (37.1 months) vs our SATS (54.4 and 33.4 months, respectively). Two American studies of AD reported a shorter time spent in NHs by almost 1 year, median of 33 and 38.57 months, respectively [18, 19], which might be explained by differences between health care systems. In most countries of northern Europe, NHP is predominantly based on the individual’s need for care, which is almost exclusively publicly funded and covers the entire population, irrespective of income or insurance [28]. This was supported by our finding that the level of education did not affect survival time in NHs. Social-service systems that ease the family members’ economic burden of care can lead to a longer time spent in NHs.

Here, males living with a family member spent a shorter time (by ~2–2.5 years) in NHs than did the other participants, after controlling for sex, age, disease severity, and concomitant medications; hence, the usually shorter lifespan of males cannot explain this observation. A large number of previous studies of dementia investigated separately the potential effects of sex and living status on community-based service utilization; in contrast, analyses of the interaction term are scarce. We reported previously a longer time (mean of 6 months) to use of home-help services [14] and a lower risk of NHP [16] among male AD patients living with family vs the corresponding females. These differences are probably explained by the informal care provided by female spouses, who are more likely to provide care for a longer period than male caregivers [29]. Moreover, the service providers may be less alert to the needs of female caregivers regarding formal help because they assume that these mostly older women can manage the care by themselves [30]. Younger females may be more educated, less committed to caring for relatives, and more assertive; thus, the demand for formal care and NHs may increase in the future.

Users of antihypertensive/cardiac therapy or anxiolytics/sedatives/hypnotics exhibited a shorter life expectancy (by about 1 year) in our multivariate models. Cardiovascular disorders are well-known risk factors for increased mortality in NH residents with dementia [20, 31]. The SATS patients who used antihypertensive/cardiac therapy were older and cognitively better at NHP, which might explain the similar age at death compared with nonusers. Furthermore, the MMSE scale was not adapted to measure altered executive function and mental speed, which are usually related to vascular pathology and might be expected in users of antihypertensive/cardiac therapy. Tests that evaluate these abilities specifically might have shown a greater cognitive impairment. Users of anxiolytics/sedatives/hypnotics were also older at NHP and had a similar age at death as the nonusers. A study of NH residents with dementia showed that the use of anxiolytics had a positive effect on basic ADL [32], which might postpone NHP. Recently, we found an association between the use of anxiolytics/sedatives/hypnotics and a longer survival time [17]. Improved sleep may lead to a more favorable outcome in AD [33] and might be an additional reason for delayed NHP.

Cognitive ability at NHP or its rate of progression did not influence survival time in NHs in the current study, which is in accordance with most previous studies of mortality in NH residents with dementia [20, 34], but not with all [35]. Consistently, cognition was not an important predictor of use of home-help services [14, 36] or time to NHP [16, 37]. Rather, a lower cognitive status was associated with fewer hours of home help [14]. This noteworthy observation suggests that the individuals’ cognitive ability was not considered when deciding the amount or level of community-based care needed, and that the recipients and family members might have been unable/unwilling to request additional help. The services provided must be better tailored to the requirements of persons with cognitive impairment.

In contrast, basic ADL (but not IADL) were an independent predictor of survival time in NHs in the present study. Worse basic ADL at NHP, a longer time between AD diagnosis and NHP, and, thus, a shorter time spent in NHs exhibited significant relationships, indicating that a longer period of care in the recipient’s home might reduce survival time in NHs. However, the burden of care for demented individuals with loss of many basic ADL must also be considered; this issue was not addressed in the SATS. Functional disability has been reported as an important predictor of mortality in many studies of NH residents with dementia [20, 34]. The time to NHP seems to be more dependent on the participants’ total ADL or IADL capacity and their rates of decline [16, 37], whereas basic ADL deficits affect life expectancy. Progression in IADL might have a lesser effect on survival vs the consequences of the loss of crucial functions during the later stages of AD, which may lead to poor personal hygiene, malnutrition, incontinence, and falls [38]. Therefore, basic ADL might serve as an indicator of the remaining life-span in end-stage dementia.

Age was not a significant predictor of survival time in NHs in our multivariate models, suggesting that the remaining factors had a stronger effect. Furthermore, the amount of home-help services or adult day care at NHP, or the annual change in these services did not predict time spent in NHs. This indicates that community-based services cannot reduce the AD patients’ survival time in NHs and supports our finding that cognitive status was not related to service utilization. Home-help services cannot sufficiently address the needs associated with cognitive impairment, such as supervision, management of behavioral symptoms, and avoidance of dangerous situations [36]. A study of adult day-care services observed that the recipients who used more care/week had an increased risk of NHP, even after controlling for disease severity and caregiver burden, which implies that adult day care serves more as a transitional period to NHP than as a respite, and, thus, indicates shortened time to admission [39]. This information is essential for service providers and raises questions regarding the utility of the services available currently to community-dwelling people with dementia.

Here, different aspects of ChEI therapy, such as drug agent, dose, level of short-term response, or treatment duration, did not affect the survival time in NHs. A modest-sized mean improvement in cognitive ability for about 6–12 months [11, 40] and a corresponding reduced decline in ADL [41] after the initiation of ChEI therapy were described by previous AD studies. Moreover, higher ChEI doses have been associated with better longitudinal cognitive and functional outcomes [11, 13, 42], a lower volume of community-based care [14], delayed time to NHP [15, 16], and a longer life-span [17]. Taken together, the initial response to ChEI, the slowing of disease progression, and the postponement of the need for community-based services entail a 6–12 month positive shift in the disease. Hence, the unchanged length of stay in NHs observed indicates that this potential increase in life expectancy occurs in the mild-to-moderate stage of AD, when the persons are able to live in their homes.

The strengths of the SATS are its 6-month, prospective, well-organized evaluations of cognitive and functional performance and resource utilization (which is less commonly measured in most studies) after the onset of ChEI treatment. Survival time in this large cohort of “real-life” AD patients with comorbidities and concomitant medications from Swedish memory clinics has now been followed for 17 years. The community-based services in Sweden are publicly funded and, therefore, accessible to all residents, regardless of their socioeconomic status [28]. Thus, we assume that the participants were representative of the general population and that their needs for formal care reflected their actual disabilities. Similar to other long-term observational studies of AD, the SATS is not placebo controlled (because of ethical concerns) or randomized with respect to ChEI agent. Another limitation is that somatic disorders and other health events, for instance, were not recorded between the last follow-up and death, which may have affected mortality.

Very few studies of AD exclusively have investigated the residents’ length of time spent in NHs and possible predictors that might affect this parameter, probably because of the long study time that would be required. No previous studies addressed the associations between type of ChEI agent, dose and duration of ChEI therapy, and survival time in NHs; thus, additional studies are warranted. The potential effect of sociodemographic factors and interaction terms—such as living status by sex, as well as various comorbidities and medications, such as anxiolytics/sedatives/hypnotics—on time spent in NHs needs further investigation.

## Conclusions

In conclusion, critical characteristics that may influence the survival time in NHs (mean, ~4 years) were identified in this naturalistic AD study. A significant interaction effect showed that men living with a family member spent less time in NHs (by ~2–2.5 years) compared with the other patients, after controlling for several factors. The possible caregiver burden on female spouses who care for husbands with AD over several years needs attention, and probably support and respite. Worse basic ADL at NHP, but not cognitive status, were related to a shorter stay in NHs, which might mirror the magnitude of the loss of essential functions during the later AD stages and the fact that the individual’s cognitive ability was less considered at NHP. The amount of, and changes in, community-based services did not affect survival time in NHs, which indicates that these types of care are not sufficient to postpone NHP. No relationships between different aspects of ChEI treatment and time spent in NHs were detected; therefore, we suggest that the positive effects of ChEIs do not prolong the end-stage of AD or the survival time in NHs.

## Abbreviations

AD: Alzheimer’s disease
ADAS-cog: Alzheimer’s Disease Assessment Scale - cognitive subscale
ADL: Activities of daily living
APOE: Apolipoprotein E
ChEI: Cholinesterase inhibitors
CI: Confidence interval
IADL: Instrumental Activities of Daily Living scale
MMSE: Mini-Mental State Examination
NH: Nursing home
NHP: Nursing home placement
NSAIDs: Non-steroidal anti-inflammatory drugs
PSMS: The Physical Self-Maintenance Scale
SATS: Swedish Alzheimer Treatment Study
SD: Standard deviation
